# Detecting jingle and jangle fallacies by identifying consistencies and variabilities in study specifications – a call for research

**DOI:** 10.3389/fpsyg.2024.1404060

**Published:** 2024-08-30

**Authors:** Barbara Hanfstingl, Sandra Oberleiter, Jakob Pietschnig, Ulrich S. Tran, Martin Voracek

**Affiliations:** ^1^Department of Psychology, University of Klagenfurt, Klagenfurt, Austria; ^2^Department of Developmental and Educational Psychology, University of Vienna, Vienna, Austria; ^3^Department of Cognition, Emotion, and Methods in Psychology, University of Vienna, Vienna, Austria

**Keywords:** jingle fallacies, jangle fallacies, validity, meta-analysis, systematic review, specification analysis, harvest plot

## Abstract

Over the past few years, more attention has been paid to jingle and jangle fallacies in psychological science. Jingle fallacies arise when two or more distinct psychological phenomena are erroneously labeled with the same term, while jangle fallacies occur when different terms are used to describe the same phenomenon. Jingle and jangle fallacies emerge due to the vague linkage between psychological theories and their practical implementation in empirical studies, compounded by variations in study designs, methodologies, and applying different statistical procedures’ algorithms. Despite progress in organizing scientific findings via systematic reviews and meta-analyses, effective strategies to prevent these fallacies are still lacking. This paper explores the integration of several approaches with the potential to identify and mitigate jingle and jangle fallacies within psychological science. Essentially, organizing studies according to their specifications, which include theoretical background, methods, study designs, and results, alongside a combinatorial algorithm and flexible inclusion criteria, may indeed represent a feasible approach. A jingle-fallacy detector arises when identical specifications lead to disparate outcomes, whereas jangle-fallacy indicators could operate on the premise that varying specifications consistently yield overrandomly similar results. We discuss the role of advanced computational technologies, such as Natural Language Processing (NLP), in identifying these fallacies. In conclusion, addressing jingle and jangle fallacies requires a comprehensive approach that considers all levels and phases of psychological science.

## Problem outline

In recent years, there has been increased attention on jingle and jangle fallacies in psychological science (Altgassen et al., 2024; Ayache et al., 2024; Beisly, 2023; Fischer et al., 2023; Hook et al., 2015; Marsh et al., 2019; Porter, 2023). Jingle fallacies occur when two or more distinct psychological phenomena are labeled with the same name, as Thorndike (1904, p. 14) defined over 120 years ago. Kelley (1927, p. 64) later defined jangle fallacies as labeling the same phenomenon with different terms, exemplified by his use of ‘intelligence’ and ‘achievement’. Gonzalez et al. (2021) highlighted that jingle and jangle fallacies pose a significant threat to the validity of the research. These fallacies are not always explicitly labeled as such; they may also be characterized as a déjà-variable phenomenon (Hagger, 2014; Hanfstingl, 2019; Skinner, 1996).

Why do jingle and jangle fallacies emerge? In essence, Thorndike (1904) and Kelley (1927) attributed their occurrence to a vague connection between psychological theory and its operationalization in empirical studies. Recent studies have emphasized the caution needed regarding jingle-jangle fallacies due to differences in algorithms used in statistical procedures (Grieder and Steiner, 2022). Another reason that exacerbates this problem is the substantial increase in scientific research since the Second World War, which has led to an increase in the overall number of studies carried out. However, as scientific knowledge continues to expand, there is an increasing need for its systematic organization and categorization. Without adequate systematization, the risk of poorly aligned parallel fields and trends operating independently increases, resulting in a disjointed theoretical landscape lacking overarching theories or paradigms. Finally, efficient progress is hindered by undetected inconsistencies in empirical evidence. Despite the long-standing knowledge of jingle and jangle fallacies, effective strategies to prevent psychological science from encountering these issues have not yet been developed.

In the 1970s, several solutions emerged to address the lack of systematization in scientific findings, with the development of review and meta-analytical approaches, albeit without explicit reference to jingle or jangle fallacies. According to Shadish and Lecy (2015), meta-analysis is considered “one of the most significant methodological advancements in science over the past century” (p. 246). Notably, Gene V. Glass focused on psychotherapy effects, Frank L. Schmidt emphasized psychological test validity, and Robert Rosenthal aimed to synthesize findings on interpersonal expectancy effects, all of whom contributed significantly to the development of meta-analysis (Shadish and Lecy, 2015).

While the practice of summarizing single studies in reviews and meta-analytical procedures has become common and well-accepted, several problems have become apparent: Systematic reviews and meta-analyses, while valuable, are not immune to bias and fail to detect jingle or jangle fallacies. Despite several initiatives like the PRISMA statement (Page et al., 2021) or meta-analysis reporting standards (MARS; Lakens et al., 2017), they still lack quality criteria (Glass, 2015; Pigott and Polanin, 2020) or ignore the influence of methodologies on the result (Elson, 2019). Some biases are extremely difficult to control, as, for example, those caused by scientists themselves (Hanfstingl, 2019; Wicherts et al., 2016) or by operationalization variances (Simonsohn et al., 2020; Steegen et al., 2016; Voracek et al., 2019). Furthermore, as with single studies, without transparency and free access to each point of the research process, reproducibility is not given (Maassen et al., 2020; Polanin et al., 2020). In sum, current review and meta-analytical approaches fail to uncover jingle or jangle fallacies.

## Approaches for detecting and preventing jingle and jangle fallacies

Essentially, we need not only programs to systematize empirical evidence and knowledge but also strategies to detect and prevent jingle and jangle fallacies, ideally combining single-study analyses at the meta-level. To address these challenges, we explore several potentially beneficial approaches. One such approach involves the systematization not only of results but also of theoretical backgrounds, methodological approaches, study designs, and outcomes. This provides, for example, specification curve analysis developed by Simonsohn et al. (2020). The procedure delineates all reasonable and debatable choices and specifications for addressing a research inquiry at the single-study level. These specifications must (1) logically examine the research question, (2) be expected to maintain statistical validity, and (3) avoid redundancy with other specifications in the array. Steegen et al. (2016) introduced the multiverse analysis concept, offering additional plotting alternatives as a similar approach. Voracek et al. (2019) combined these approaches at a meta-analytical level, revealing the range of formally valid specifications, including theoretical frameworks, methodological approaches, and researchers’ degrees of freedom. They distinguish between internal (“which,” e.g., the selection of data for meta-analysis) and external (“how,” e.g., the methodology of data meta-analysis) factors. Identifying reasonable and formally valid specifications is considered a crucial first step in gaining an overview of which aspects and perspectives of a psychological phenomenon have already been empirically investigated.

Detecting and preventing jingle and jangle fallacies requires considering as many studies as possible to obtain a comprehensive overview. However, addressing the relatively strict and sometimes poorly justified inclusion and exclusion criteria in systematic reviews and meta-analyses presents a further challenge (Uttley et al., 2023). The current practice of setting rigid criteria in meta-analyses may be overly stringent, leading to the exclusion of valuable but non-quantifiable studies. Several approaches have less strict inclusion criteria, such as the harvest plot (Ogilvie et al., 2008). The harvest plot considers studies by graphical displays that otherwise would be excluded due to missing quantifiable data or effect estimates for meta-analyses, plotting the quality, the study design variances, differences of included variables, and outcome information of the studies. Foulds et al. (2022) described harvest plots as an exploratory method that allows for grouping outcomes, including non-parametric statistical tests, studies without effect sizes, and depiction of biases within studies. Comparing the results of a meta-analysis and a harvest plot analysis derived from the same study corpus reveals that the harvest plot approach allows for the inclusion of a significantly higher number of studies in the analysis (Foulds et al., 2022, Table 3). Accordingly, techniques like harvest plots play a vital role in expanding the scope of analyzed findings, which is crucial for achieving a comprehensive understanding of studies on a specific phenomenon.

## Implementing jingle and jangle detectors

**Figure 1:**
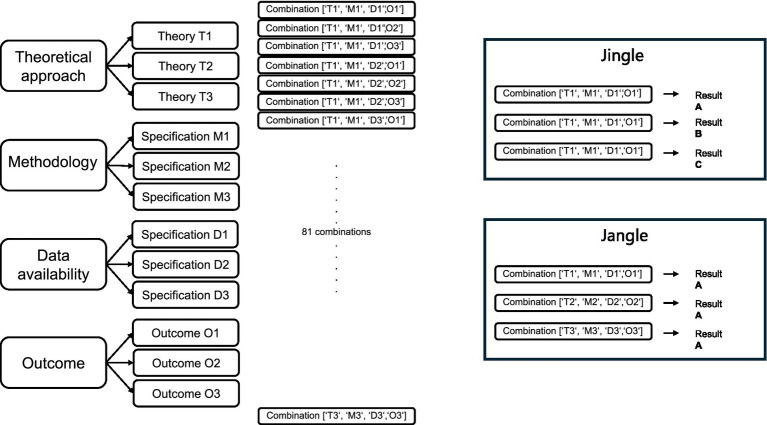
Exemplary specifications derived from theory, methodology, data availability, and results.

As described, various useful approaches effectively structure and organize studies on a psychological phenomenon. But how can we detect potential jingle and jangle fallacies? Harvest plots summarize the findings of studies based on their suitability of study design, quality of execution, variance-explaining dimensions (such as gender and race), and outcomes quality (e.g., behavioral, self-reports). The plots offer descriptive representations and provide an overview of previously investigated results. Empty lines indicate missing data for known combinations of variables or specifications (see, e.g., Ogilvie et al., 2008, Figure 1). However, they still lack a combinatorial approach, as suggested by Simonsohn et al., 2020 or Voracek et al. (2019). After implementing the permutational aspect on specifications, a jingle fallacy detector could be based on the idea that, in the presence of a jingle, the same specifications would lead to different results. Conversely, jangle fallacy detectors would operate *vice-versa* and indicate jangle if different specifications yield overrandomly similar results. Figure 1 illustrates how combining different theoretical approaches, methodologies, data availabilities, and outcomes can help identify potential jingle and jangle fallacies.

Thus far, two promising approaches have concentrated on detecting jingle and jangle fallacies at a taxonomic level. Larsen and Bong (2016) presented six different so-called construct identity detectors for literature reviews and meta-analyses, applying different natural language processing algorithms. Wulff and Mata (2023) provided a solution in a preprint, utilizing GPT at the level of personality taxonomies to analyze the items and their scale assignments in the international personality item pool (IPIP; Goldberg et al., 2006). Since GPT is based on Natural Language Processing, it is well-suited to detect jingle and jangle fallacies within taxonomic approaches. However, reliance on taxonomies alone is insufficient for detecting jingle and jangle fallacies in psychological science. We understand psychological phenomena through theories, operationalized with concepts, constructs, and methodologies, and measured through physiological and behavioral data, self-reports, and external reports. Empirical data hinges on these interconnected elements alongside methodologies and study designs (Uher, 2023). Therefore, to detect jingle and jangle fallacies, we must consider all these levels and phases of psychological science.

## Conclusion

**Figure 2:**
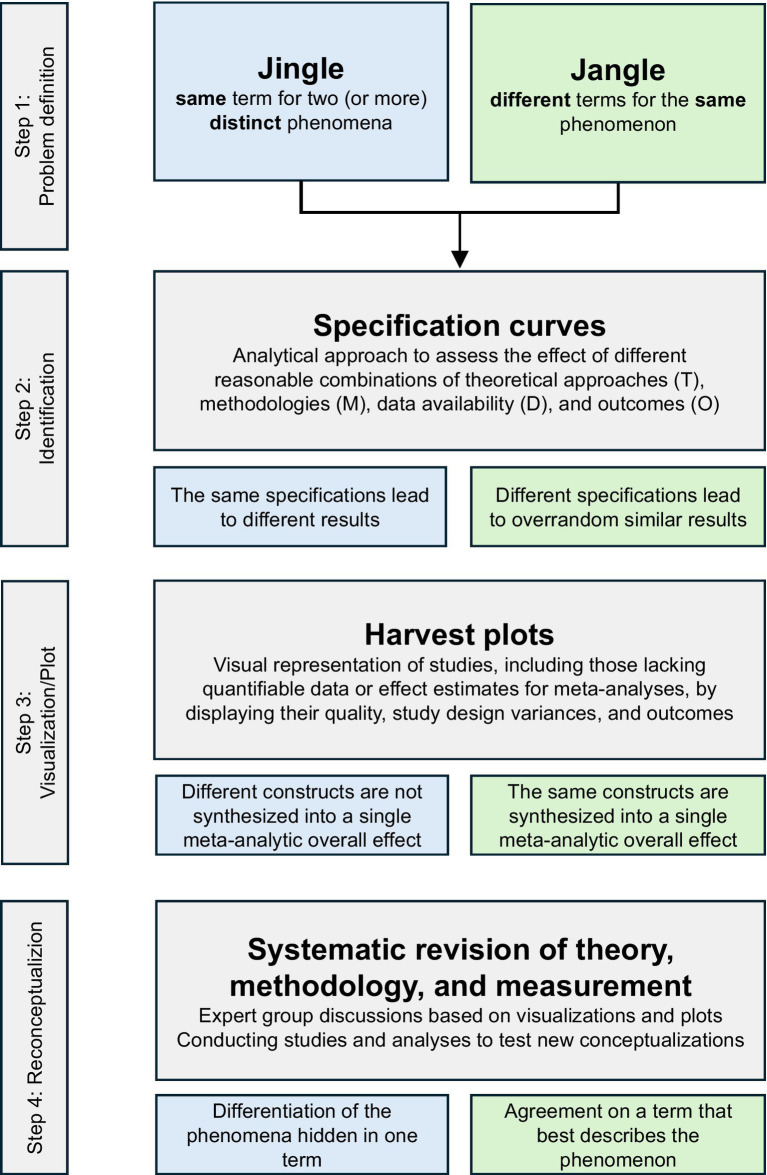
Process for systematically revising jingle and jangle fallacies.

The growing attention to jingle and jangle fallacies in recent years underscores their significance in psychological science, posing a threat to validity and often going unrecognized. These fallacies, originally defined by Thorndike (1904) and Kelley (1927), emerge due to vague connections between theoretical concepts and empirical operationalizations but also have pure computational roots (Grieder and Steiner, 2022). Developments like meta-analyses and systematization through reviews help to systematize knowledge, but these practices are not immune to biases and limitations (Uttley et al., 2023) and do not detect jingle and jangle fallacies – such detectors are not yet developed. These detectors need to consider all levels and phases of psychological science, from theoretical frameworks to methodological approaches and study designs, called study specifications (Simonsohn et al., 2020). Additionally, flexible inclusion criteria for considered studies and new computational approaches, as conducted by Larsen and Bong (2016) or Wulff and Mata (2023) are needed. Ultimately, addressing jingle and jangle fallacies requires a concerted effort across the scientific community, incorporating diverse theories, perspectives, and methodologies. Simply defining the problem – finding one term for multiple phenomena (jingle) or different terms for the same phenomenon (jangle) – is insufficient. A systematic revision of jingle and jangle fallacies, achieved through discussion and analysis of detected instances is essential, as outlined in Figure 2.

## Data Availability

The original contributions presented in the study are included in the article, further inquiries can be directed to the corresponding author.
